# Spatial determinants of excess all-cause mortality during the first wave of the COVID-19 epidemic in France

**DOI:** 10.1186/s12889-021-12203-8

**Published:** 2021-11-24

**Authors:** Hugo Pilkington, Thierry Feuillet, Stéphane Rican, Jeanne Goupil de Bouillé, Olivier Bouchaud, Johann Cailhol, Hélène Bihan, Pierre Lombrail, Chantal Julia

**Affiliations:** 1 Département de Géographie, Université Paris 8 Vincennes-Saint-Denis, UMR7533 Ladyss, 2 rue de la Liberté, F-93526 Saint-Denis, France; 2 Sorbonne Paris Cité Epidemiology and Statistics Research Center (CRESS), Inserm U1153, Inra U1125, Cnam, Paris 13 University, Nutritional Epidemiology Research Team (EREN), Bobigny, France; 3 Département de Géographie-Aménagement, Université Paris Nanterre, UMR7533 Ladyss, 200 avenue de la République, F-9200 Nanterre, France; 4 Infectious and Tropical Diseases Department, Avicenne Hospital (AP-HP), Bobigny, France; 5 LEPS, Laboratoire Educations Pratiques Santé, UR3412, Université Sorbonne Paris Nord Bobigny, Villetaneuse, France; 6 Endocrinology, Diabetology and Metabolic diseases Department, Avicenne Hospital (AP-HP), Bobigny, France; 7 Public Health Department, Avicenne Hospital (AP-HP), Bobigny, France

**Keywords:** COVID-19, Mortality, Socioeconomic factors, Delivery of health care, Deprivation, Inequality

## Abstract

**Background:**

The first wave of the COVID-19 pandemic in France was associated with high excess mortality, and anecdotal evidence pointed to differing excess mortality patterns depending on social and environmental determinants. In this study we aimed to investigate the spatial distribution of excess mortality during the first wave of the COVID-19 pandemic in France and relate it at the subnational level to contextual determinants from various dimensions (socioeconomic, population density, overall health status, healthcare access etc.). We also explored whether the determinants identified at the national level varied depending on geographical location.

**Methods:**

We used available national data on deaths in France to calculate excess mortality by department for three age groups: 0–49, 50–74 and > 74 yrs. between March 1st and April 27th, 2020. We selected 15 variables at the department level that represent four dimensions that may be related to overall mortality at the ecological level, two representing population-level vulnerabilities (morbidity, social deprivation) and two representing environmental-level vulnerabilities (primary healthcare supply, urbanization). We modelled excess mortality by age group for our contextual variables at the department level. We conducted both a global (i.e., country-wide) analysis and a multiscale geographically weighted regression (MGWR) model to account for the spatial variations in excess mortality.

**Results:**

In both age groups, excess all-cause mortality was significantly higher in departments where urbanization was higher (50–74 yrs.: β = 15.33, *p* < 0.001; > 74 yrs.: β = 18.24, *p* < 0.001) and the supply of primary healthcare providers lower (50–74 yrs.: β = − 8.10, *p* < 0.001; > 74 yrs.: β = − 8.27, *p* < 0.001). In the 50–74 yrs. age group, excess mortality was negatively associated with the supply of pharmacists (β = − 3.70, *p* < 0.02) and positively associated with work-related mobility (β = 4.62, *p* < 0.003); in the > 74 yrs. age group our measures of deprivation (β = 15.46, *p* < 0.05) and morbidity (β = 0.79, *p* < 0.008) were associated with excess mortality. Associations between excess mortality and contextual variables varied significantly across departments for both age groups.

**Conclusions:**

Public health strategies aiming at mitigating the effects of future epidemics should consider all dimensions involved to develop efficient and locally tailored policies within the context of an evolving, socially and spatially complex situation.

**Supplementary Information:**

The online version contains supplementary material available at 10.1186/s12889-021-12203-8.

## Background

Faced with the epidemic of COVID-19, French authorities (like in many other countries in Europe and around the world) initiated a 55-day long nationwide lockdown on March 17th that was lifted progressively on May 11th, 2020, in a bid to quell the risk to the general population and faced with the very real possibility of a systemic failure in the country’s hospital system. Questions have been raised about the extent of COVID-19 mortality during this period. Government health agencies have produced regular data in order to estimate the actual impact on the population of deaths from the disease and thus its overall impact on mortality and ensuing geographic variations. Moreover, concerns have been raised over the social distribution of mortality, early data suggesting that more vulnerable populations were not only more prone to being infected by Sars-Cov2, but also were more at risk of a fatal outcome [1].

All-cause nationwide mortality in mainland France was 238,271 deaths over the period stretching from January 1st to April 30th 2020, significantly greater than for the same period in both 2019 and 2018, highlighting the burden associated with the COVID-19 pandemic^﻿2^. Between March 1st and April 30th, mortality rates were much higher in the urban densely populated administrative region of Ile-de-France (i.e. Paris and surroundings, + 90%) and in the region where the largest outbreak of the disease occurred first (in north-eastern France called Grand Est, + 55%) compared to the same period in the two preceding years [2]. These variations in the overall spatial pattern of mortality during the first wave of the pandemic have been linked to specific events and/or known exacerbating factors in the dynamics of an infectious disease epidemic, but questions have been raised over environmental and social determinants beyond these [3, 4]. As detailed data on COVID-19 deaths is still to be consolidated nationally, investigating the overall (i.e. all-cause) mortality during the first wave of the COVID-19 pandemic and the determinants of spatial differences in death rates that may be associated directly or indirectly with COVID-19 may be used as a valid proxy. Thus, understanding the spatial distribution of overall mortality may help us grasp the geographic scope of the contextual disease-specific determinants associated with COVID-19 mortality.

In this paper, we seek to describe the spatial distribution of overall mortality at the department (sub-national) scale and relate it to contextual determinants from various dimensions (socioeconomic, population density, overall health status, healthcare access etc.). A further objective was to explore whether the determinants identified at the national level varied across territories, i.e. at the sub-national level.

## Methods

### Data

We used national data on deaths in France according to the department where deaths occurred (*N* = 119,546), available from INSEE (the French National Institute for Statistics and Economic Studies). Mainland France has a population of 65.3 million inhabitants and roughly one-fifth (21%) of the population is aged over 65. Mortality rates in the general population have been slightly increasing over 2010–2019 (from 8.6 to 9.2 per 1000 population), mostly due to an ageing population [5].

Deaths recorded for each municipality from March 1st to April 27th, 2020 were aggregated at the department level and updated starting March 25th, 2020 (there are 34,839 municipalities known as “communes” grouped into 96 departments in mainland France; these are in turn grouped into 13 administrative regions such as “Ile de France” or “Grand Est”). This period coincides with the initial rise and subsequent peak of the Spring epidemic in France. We used data by department because they are the most robust consolidated data available to date. It should be noted that deaths are usually corrected to be recorded at the municipality of residence, but this is not yet the case for 2020. We also used data from deaths recorded at the department of residence for the two preceding years, 2019 (*N* = 95,867) and 2018 (*N* = 104,006).

### Definitions

#### Main outcome measure: excess mortality March 1st to April 27th, 2020

Our main outcome measure was excess mortality, defined as the relative difference (in percentage) between deaths over the study period compared to deaths over the same period in 2019 and 2018, a two-year period recommended by INSEE for calculating excess mortality for the COVID-19 pandemic in France [6]. We were also able to calculate excess mortality by department for the following age groups: 0–49, 50–74 and > 74 yrs. The data were provided for these age groups by INSEE, as the 50–74 and > 74 yr age groups are those most at risk for severe COVID-19 illness and death [1].

#### Department-level contextual variables

We initially selected 15 variables available at the department level that represent four dimensions that we hypothesized may be related to overall mortality at the ecological level: 1) COVID-19 associated chronic diseases (three variables), 2) healthcare supply (three variables), 3) social deprivation (four variables) from a previously validated French deprivation index, “FDEP” [7], and 4) urbanization (five variables). For the “healthcare supply” and “urbanization” dimensions, principal components analyses (PCA) were performed to obtain up to two independent variables representing the overall dimension rather than including each variable in the models, thereby limiting multicollinearity within a single dimension. A detailed presentation (both of all the variables and of the resulting selected ones) is available in the supplementary material (Additional file 1, Table 1). The final 7 contextual variables for our analysis were thus: COVID-19 related morbidity (referred to hereafter as “morbidity”, these are long-term conditions covered by the French national health insurance coverage), supply of primary healthcare providers (general practitioners and nurses), supply of pharmacists, FDEP, deprivation heterogeneity, urbanization, and work-related mobility.

#### Analysis

First, we described excess mortality by age group (0–49, 50–74 and > 74 yrs) and department in France over the study period. We then modelled excess mortality by age group for our contextual variables at the department level. We conducted both a global (i.e., country-wide) analysis and a geographical analysis to account for the spatial variations in excess mortality. *P*-values < 0.05 were considered statistically significant.

#### Modelling strategy

Associations between excess mortality and the seven contextual variables were modelled following a twofold strategy. First, a global ordinary least square regression model was computed to assess mean associations over the entire country. Second, a multiscale geographically weighted regression (MGWR) model was estimated to account for spatial non-stationarity of regression coefficients, i.e., spatial heterogeneity of relationships across space. This methodology has been described in detail elsewhere [8–10] and we have included a detailed description in the supplementary material (Additional file 1, Box 1).

All the MGWR models were estimated using the R package GWmodel [11]. All analyses were performed using R (version 4.0.3).

## Results

**Fig. 1:**
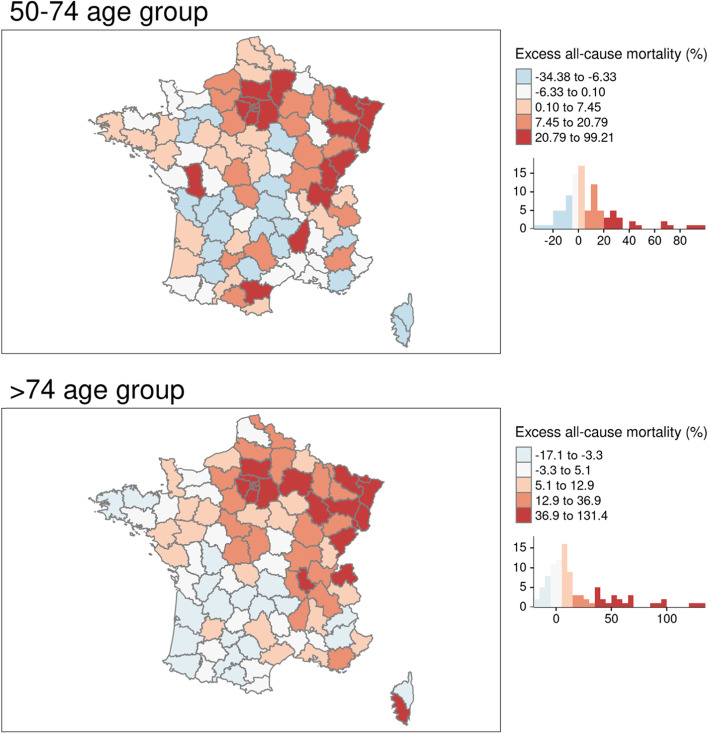
Excess all-cause mortality (mainland France) by age group over the study period compared to 2018–19. Source of maps: authors

Over the study period, overall excess mortality for the 0–49 age group ranged from − 56.52 to + 114.29% (mean = − 5.59%) at the national level. However, no clear geographical pattern was apparent, so our analyses were limited to descriptive geographical analyses (data not shown here but available upon request). For all three age groups, there were departments with negative excess mortality, meaning that there had been a reduction in all-cause mortality over the study period. An overall excess mortality was observed for the 50–74 and > 74 age groups, albeit with high variability depending on the department. Half of all departments experienced excess mortality levels over 4% for both age groups (50–74: 4.15%; > 74: 7.69%) and on average excess mortality was more pronounced for the > 74 age group (m = 19.79%) than for the 50–74 age group (m = 9.62%). Excess mortality was apparent for the > 74 age group for most departments in north-eastern France. Excess mortality was highest in the most deprived mainland French department (Seine-Saint-Denis, containing many of north-eastern suburbs of Paris) for both age groups: + 99.21% for the 50–74 age group and + 131.43% for the > 74 age group whereas it was lowest in Corse-du-Sud (the southern part of the island of Corsica: − 34.38% for the 50–74 age group) and Tarn-et-Garonne, a sparsely populated department in south-western France: − 17.14% for the > 74 age group (Fig. 1).

**Fig. 2:**
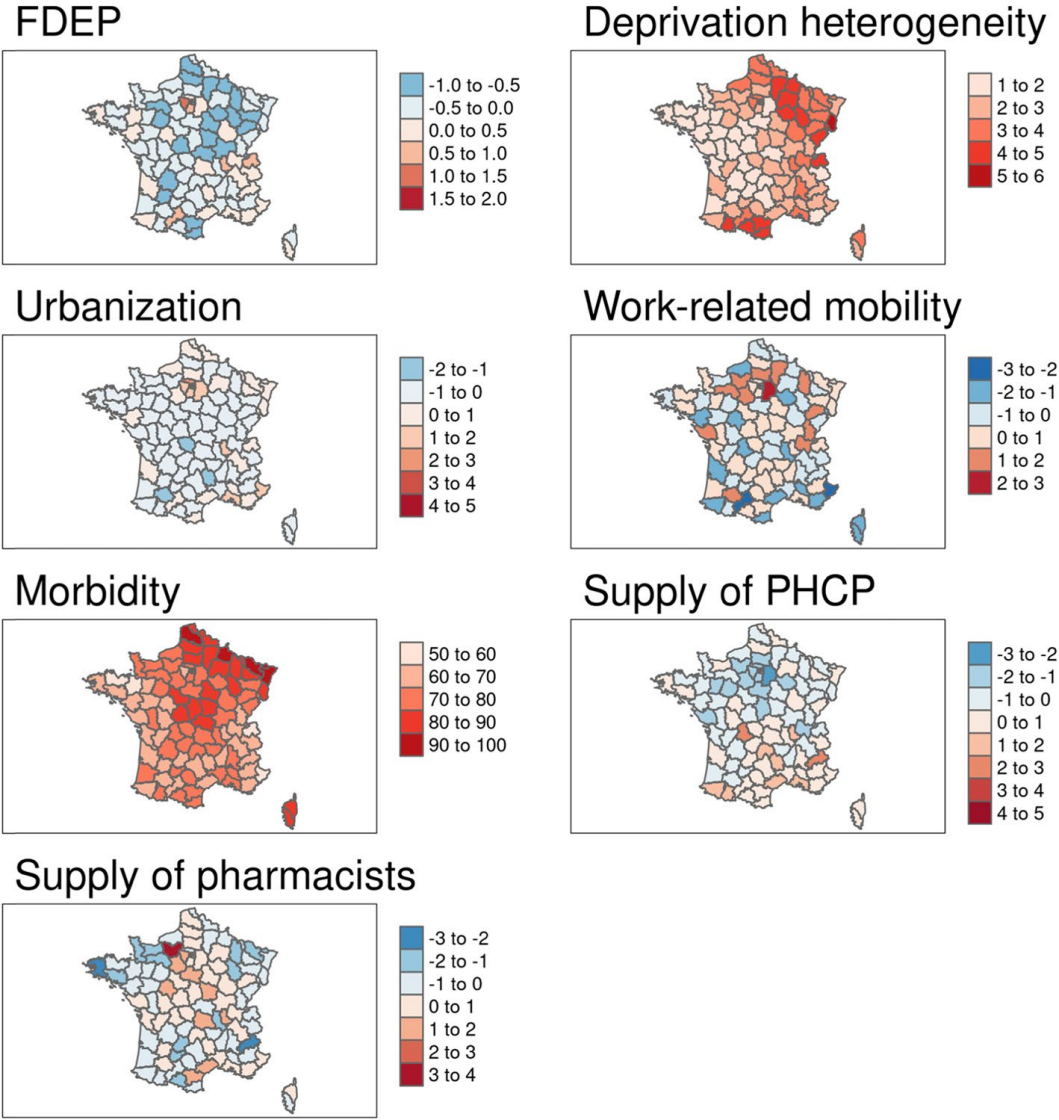
Spatial distribution of (unadjusted) selected contextual variables associated with excess all-cause mortality. Source of maps: authors. Notes: The maps represent standard deviations with a mean = 0, except for morbidity, in %). FDEP=French deprivation index; Supply of PHCP = supply of primary healthcare providers

In the global multivariable analyses (i.e. the OLS models), excess mortality was positively associated with urbanization and negatively associated with the supply of primary healthcare providers for both age groups. A one-point increase in urbanization yielded a > 15 percentage point increase in excess mortality for both age groups; a one-point increase in the supply of primary healthcare providers reduced excess mortality by > 8 percentage points. Furthermore, work-related mobility was positively associated with excess mortality and the supply of pharmacists was negatively associated with excess mortality in the 50–74 age group. In addition, FDEP, deprivation heterogeneity and morbidity were all positively associated with excess mortality for the > 74 age group. (Table 1, Fig. 2).

**Table 1:** Contextual variables associated with excess all-cause mortality in mainland France by age group (OLS models)

	50–74 years old			> 74 years old		
	β Coefficient ^a^	95% Confidence Interval	*p*-value	β Coefficient	95% Confidence Interval	*p*-value
FDEP	6.56	[−4.34; 17.46]	0.24	15.46	[0.86; 30.07]	0.041
Deprivation heterogeneity	3.03	[−0.04; 6.09]	0.056	5.83	[1.72; 9.94]	0.007
Urbanization	15.33	[10.95; 19.70]	< 0.001	18.24	[12.38; 24.10]	< 0.001
Work-related mobility	4.62	[1.68; 7.57]	0.003	3.59	[−0.36; 7.54]	0.078
Morbidity	0.24	[−0.18; 0.67]	0.26	0.79	[0.22; 1.36]	0.008
Supply of primary healthcare providers	−8.10	[−11.47; −4.73]	< 0.001	− 8.27	[− 12.78; −3.76]	< 0.001
Supply of pharmacists	−3.70	[−6.65; −0.76]	0.016	−1.73	[− 5.68; 2.21]	0.39
*R*^2^	0.70			0.71		
Adjusted R^2^	0.68			0.68		
AICc	786.59			842.76		

^a^a 1-point increase in FDEP yields a 6.56-point increase in all-cause excess mortality in the 50–74 yrs. age group

**Fig. 3:**
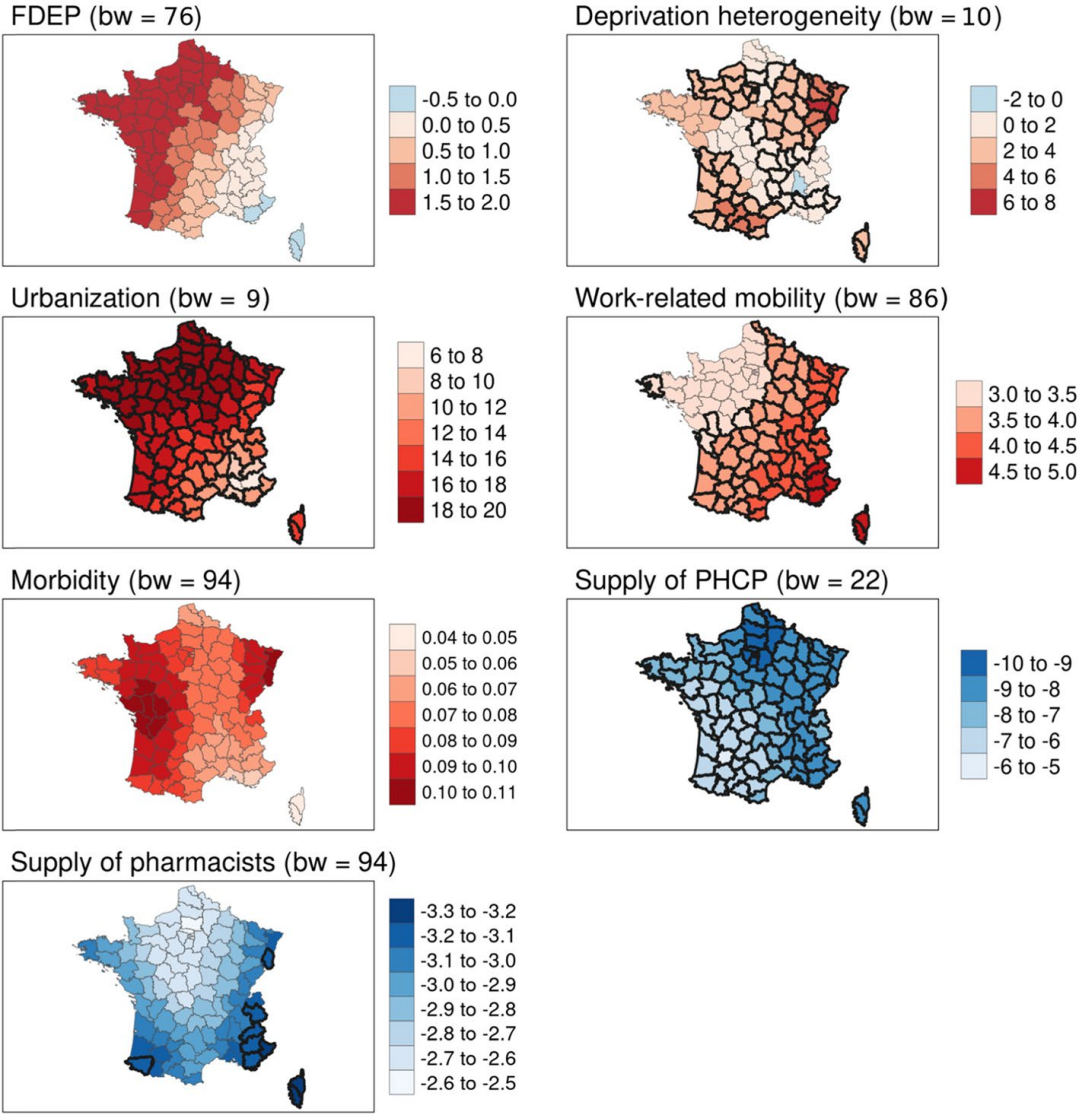
Geographically weighted associations of contextual variables with excess all-cause mortality for the 50–74 age group. Source of maps: authors. Notes: The maps show critical t-values > |2.5| in bold. FDEP=French deprivation index; Supply of PHCP = supply of primary healthcare providers; bw = bandwidth

**Fig. 4:**
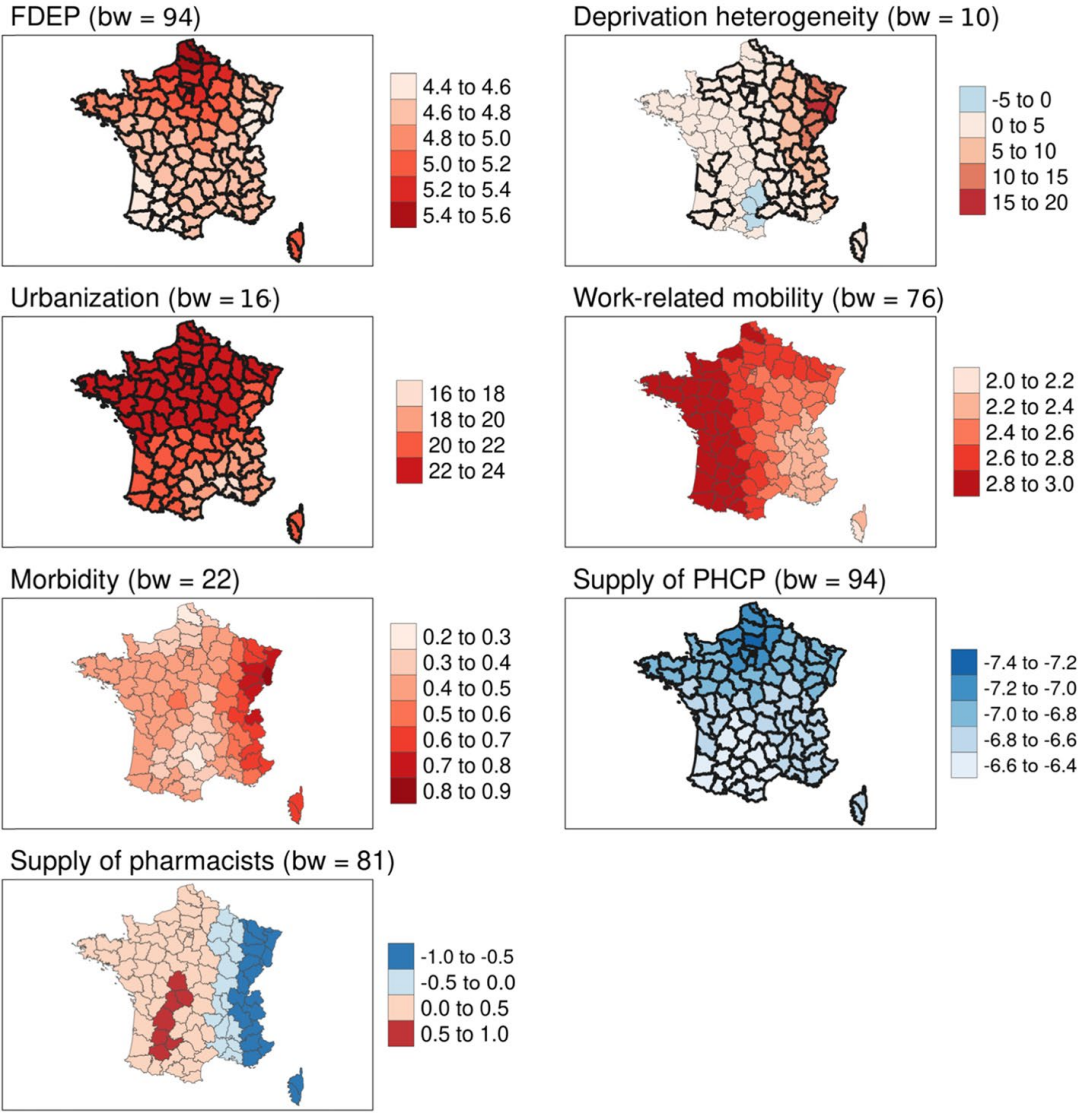
Geographically weighted associations of contextual variables with excess all-cause mortality for the > 74 age group. Source of maps: authors. Notes: The maps show critical t-values > |2.5| in bold. FDEP=French deprivation index; Supply of PHCP = supply of primary healthcare providers; bw = bandwidth

Multivariate local analyses (i.e. the MGWR models) showed better measures of goodness of fit than the OLS models for both age groups (adjusted R^2^: 0.68 for the OLS models vs 0.73 and 0.78 for the MGWR models), as well as smaller corrected AIC, indicating a better relative quality (Table 2). Results indicate that the associations between excess mortality over the study period and the contextual variables varied in intensity over space, reflecting the spatial heterogeneity of the relationships. Spatial non-stationarity affected deprivation heterogeneity significantly for both age groups, with the highest values in southern and eastern France, where a one-point increase in this variable resulted in a 5 to 6 percentage point increase in excess mortality rates for the 50–74 age group and an increase of 15–20 percentage points in the > 74 age group. Furthermore, for the 50–74 age group, work-related mobility was associated with the highest increase in excess mortality (> 4.5%) in south-eastern France, including the island of Corsica. Inversely, the supply of pharmacists was significantly associated with a reduction in excess mortality in south-eastern France, the eastern department of Haut-Rhin and the southwestern department of Pyrénées-Atlantiques (> − 3.2%; Table 2, Figs. 3 and 4).

**Table 2:** Contextual variables associated with excess all-cause mortality in mainland France by age group, values by quartile (β coefficients from MGWR models)

	50–74 years old						> 74 years old					
	*bw*^*a*^	Min^b^	Q1	Median	Q3	Max	*bw*^*a*^	Min^b^	Q1	Median	Q3	Max
FDEP	*76*	−0.16	0.62	1.33	1.64	1.97	*94*	4.52	4.64	4.72	5.01	5.56
Deprivation heterogeneity	*10*	−0.39	1.56	2.21	3.02	7.22	*10*	−0.43	1.56	2.54	4.60	16.51
Urbanization	*9*	6.62	14.55	17.21	18.79	19.40	*16*	16.53	20.50	22.02	22.81	23.66
Work-related mobility	*86*	3.17	3.41	3.73	4.16	4.99	*94*	2.18	2.53	2.64	2.87	3.00
Morbidity	*94*	0.05	0.07	0.08	0.09	0.10	*22*	0.28	0.40	0.43	0.50	0.80
Supply of primary healthcare providers	*22*	−9.79	−8.55	−8.04	−7.07	−5.81	*94*	−7.23	−6.94	−6.77	−6.63	−6.48
Supply of pharmacists	*94*	−3.27	−3.05	−2.92	−2.71	− 5.58	*81*	−0.97	−0.31	0.17	0.39	0.59
*R*^2^ value	0.80						0.84					
Adjusted *R*^2^	0.73						0.77					
AICc	779.73						823.42					

^a^bw: bandwidth (number of neighbours in each parameter-specific weighting scheme)

^b^a 1-point increase in FDEP in the 50–74 yrs. age group yields a − 0.16 to 0.62 point increase in all-cause excess mortality for 25% of all departments

## Discussion

We found that excess all-cause mortality during the initial phase of the COVID-19 epidemic in France was higher for the > 74 year-old age group than the 50–74 age group, and at higher levels in north-eastern France, as has been previously described in early national reports [2]. In both the 50–74 and > 74 yrs. age groups, excess all-cause mortality was significantly higher in departments where urbanization was higher and the supply of primary healthcare providers (general practitioners and nurses) lower. Additionally, in the 50–74 yrs. age group, excess mortality was negatively associated with the supply of pharmacists and positively associated with work-related mobility, while in the > 74 yrs. age group both measures of deprivation (FDEP and deprivation heterogeneity) and morbidity were associated with excess mortality. Associations between excess mortality and contextual variables varied significantly across departments for both age groups, with higher magnitude of associations for FDEP in the east of France, for FDEP and supply of primary healthcare providers in the north of France.

### Interpretations

We show here that the contextual determinants of COVID-19 mortality vary in intensity over French departments. This means that similar factors play out differently according to the scale and the location at which they are analysed. Elsewhere, variability in the contextual determinants of COVID-19 mortality and infection has been also suggested by some authors. In France, Gaudart and colleagues have highlighted the spatial heterogeneity of in-hospital COVID-19 incidence rates at the department level, but they found no association with contextual variables such as healthcare services, economic indicators and urbanization in multivariate models [12]. Conversely, spatial analyses in Colorado have shown that population density, poverty, and unemployment, as well as underlying health conditions were associated with higher mortality during the spring wave of the pandemic [13]. Research in the Chicago and New York urban areas have suggested that while hotspots of COVID-19 in New York were mostly located in working-class and middle-income communities, those in Chicago were located in more vulnerable neighbourhoods [14]. Analyses exploring the spatial non-stationary association between social vulnerability and COVID-19 case counts in the USA have shown that the associations between housing, transportation, minority status or language and COVID-19 differed according to states [15]. However, while we found that associations were overall in the same direction with varying magnitudes of effects, the authors of that study did observe diverging effects depending on the state. This disparity in results may be due to a somewhat higher homogeneity in the distribution of comorbidities across social and ethnic groups and more importantly in access to healthcare across French departments compared to US states. At the European level, GWR analyses have shown that the association between poverty, income and total population varied in intensity according to the country, confirming the importance of exploring these associations at the local level to inform more tailored policy decision-making [16].

There are two major determinants associated with all-cause mortality during the first wave of COVID-19 in France. First, lower supplies of all primary healthcare providers (general practitioners, nurses, and pharmacists) were associated with increased mortality in our study, in both age groups, as well as with higher magnitudes of associations in the region around and to the north of Paris. Although accessibility to medical providers was promoted during the lockdown period, with measures such as facilitated access to teleconsultations (including full reimbursement by the national health insurance) [17], the absolute number of primary healthcare providers in France is currently at its lowest levels, following a secular trend limiting access to medical schools over the last decades [18]. Thus, the association between increased all-cause mortality and lower supplies of primary healthcare providers could reflect poorer health conditions of the population of these areas as well as the prevalence of COVID-19 related chronic diseases. Alternatively, during the pandemic, patients may have avoided medical care because of fear or misinformation and lower availability of primary healthcare providers (general practitioners and nurses), leading to delays in hospitalisation. Though demographic projections of the healthcare workforce predict an upward trend as of 2020, the results from our study highlight the need for improved primary healthcare accessibility, as well as a more equitable geographic distribution, especially in the event of a health emergency.

Second, our study highlights the role of urbanization. Previous research found a positive association between the French deprivation index we have used in this research and overall mortality in France at the commune level [7]. Furthermore, they found a larger difference between standardised mortality ratios in all-cause mortality between the least and most deprived urban communes, compared to rural communes. In part, this reflects the spatial concentration of risk factors and the disparity in levels of socioeconomic deprivation that are on average greater in urban areas. This also reflects those characteristics of urban areas most often associated with the severity of infectious disease outbreaks that have spurred multiple modifications in the urban landscape over the decades [19]. The current pandemic could lead to modifications in the ways urban areas are organized, from work-related mobility to the modifications in the overall population distribution [20]. For instance, some effects both of the epidemic and of the subsequent lockdowns after the Spring period of the epidemic studied here have already been observed in population mobility, such as increased mobility from urban centres to more rural areas, at least for populations with higher incomes and those who are able to work from home. Results from our study indicate that such a strategy could be hazardous, as a higher work-related mobility was significantly associated with increased all-cause mortality for the 50–74 yrs. age group.

Our results show how the various dimensions that were explored were highly intertwined not only at the national level, but even more importantly within more deprived territories, highlighting a probable syndemic at play in the context of COVID-19 [21]. The concept of a syndemic is used to describe the coexistence of multiple conditions within a population, contributing and resulting at the same time from inequalities in health [22]. Initially applied to HIV and chronic conditions, the concept is aptly applied here to describe the interplay between social deprivation, a higher prevalence of chronic conditions, higher levels of urbanization and lower availability of primary healthcare providers. These intertwined factors all exacerbate the disease burden and have additive negative effects by interacting with existing NCDs and challenging social conditions [23]. The syndemic framework has been applied to describe the interplay of factors impacting predominantly black communities in the USA, suggesting an underlying role of systemic (or structural) racism. Indeed, multiple studies in the USA have shown a higher burden of COVID-19 in minority groups, also highlighting a clustering of risk factors in minority communities in terms of social vulnerabilities and health status [24–27]. Social and health disparities are somewhat similar between France and the USA, and we cannot directly rule out underlying mechanisms of inequalities based on ethnicity at play in France. This is supported by the fact that there was an increase in all-cause mortality for those individuals born on the African continent (+ 114%) or in Asia (+ 91%) in March and April of 2020 [28]. Further studies should address the issue in greater detail.

### Strengths and limitations

Our study’s strengths include the use of consolidated data at the national level for all-cause mortality. Studies using data at the individual level have highlighted the role of social determinants in the onset, spread and severity of COVID-19 [12, 29]. The consistency we have observed between our results at the department level (an aggregated spatial level) and studies at the individual level thus validates the methodology we have used to investigate the contextual determinants of health and has limited the magnitude of potential ecological bias in our study. Moreover, we were able to investigate the multiple dimensions of the contextual determinants of health potentially associated with excess mortality during the period, thus providing important insights into the relative importance of each of these factors. To our knowledge, no study has investigated the spatial determinants of all-cause excess mortality during the first wave with such a large number of complementary dimensions, including both vulnerabilities at the population level (morbidity, deprivation, mobility) and at the environmental level (urban density, healthcare supply).

Our study has several limitations. First, its ecological nature does not allow for inferences at the individual level directly or for causality, and therefore should be interpreted with caution. For instance, there are no data on the prevalence of obesity within the general population. Second, INSEE provided accurate aggregated data, but deaths were recorded at the place of event, rather than the place of residence, to ensure rapid dissemination of results and follow-up of the dynamics of the epidemic. Though at the national level we may hypothesize that only a limited number of deaths were recorded in a neighbouring department, this may not be the case in the densely populated urban area surrounding Paris. Also, a larger number of deaths may have been recorded within Paris where the hospital density is higher than in the rest of the region, rather than at the actual department of residence. Second, though estimates for the various determinants were derived from nationally available statistics from the five previous years, some estimates may not be accurate in the context of the current pandemic. Third, our analysis here is limited to the first wave in France of the COVID-19 because we had consolidated data for that period; also, the contextual variables that we took into account in our analyses were specifically targeted to include elements related to the living conditions during that first lockdown period. As these restrictive conditions were subsequently relaxed, we cannot rule out that the dynamics would be different during the second (and now third) waves.

## Conclusions

Our results highlight the role of contextual determinants of mortality during the first wave of COVID-19 in France, with combined effects of both population (morbidity, deprivation) and environmental (urbanization, healthcare supply) factors. Furthermore, we find spatial heterogeneity depending on the local context. Public health strategies aiming at mitigating the effects of future epidemics should consider all dimensions involved to develop efficient and locally tailored policies within the context of an evolving, socially and spatially complex situation [30].

## Supplementary Information

**Additional file 1.**

## Data Availability

All the data used in this research are freely available and in the public domain. Daily counts of deaths by department and year in France used in this study are freely available at: https://www.insee.fr/fr/statistiques/4487988?sommaire=4487854. Data on the covariables referenced in the text are available upon motivated request by contacting the authors.

## Abbreviations

FDEP: French deprivation index
GWR: Geographically weighted regression
INSEE: French National Institute for Statistics and Economic Studies
MGWR: Multiscale geographically weighted regression
