# Supplementary material for: Spatial determinants of excess all-cause mortality during the first wave of the COVID-19 epidemic in France
Source: BMC Public Health. 2021 Nov 24;21:2157. doi: 10.1186/s12889-021-12203-8 (PMC8611998; doi:10.1186/s12889-021-12203-8)
Supplement: Supplementary file 1 — Additional file 1. [file 12889_2021_12203_MOESM1_ESM.docx]

**Supplementary data to: Levels, characteristics, and spatial determinants of excess all-cause mortality during the first wave of the COVID-19 epidemic in France (March 1^st^ to April 27^th^, 2020)**

**Table 1. Detailed presentation of original variables and resulting contextual variables for analysis included in this study**

| **Dimension** | **Variables included in the model** | **Initial variables combined** | **Description of the variable included in the model** |
| --- | --- | --- | --- |
| **COVID-19 related diseases**  **PCA**   - % ALD CVD, - % ALD Diabetes - % ALD Hypertension | Morbidity | Axis 1 of PCA  (+) % ALD CVD,  (+) % ALD Diabetes  (+) % ALD Hypertension | Overall prevalence of CVD, Diabetes and Hypertension. Higher scores = higher prevalence |
| **Healthcare supply**  **PCA**   - Density general practitioners - Density nurses - Density pharmacists | Supply of primary healthcare providers | Axis 1 of PCA  (+) Density general practitioners  (+) Density nurses  (+) Density pharmacists | Overall density of all practitioners  Higher scores = higher density of primary healthcare providers |
|  | Supply of pharmacists | Axis 2 of PCA  (+) Density pharmacists | Density of pharmacists specifically  Higher scores = higher density of access to pharmacists |
| **Urbanization PCA**   - Over occupation of housing - Percentage population living in an urban center - Population density (log) - Percentage population using public transportation - Percentage population working outside its residential area | Urbanization | (+) Over occupation of housing  (+) % population living in an urban center  (+) Population density (log)  (+) Percentage population using public transportation  (+) % population working outside residential area | Overall urban density  Higher scores = higher urbanization |
|  | Work-related mobility | (-) % population living in an urban center  (+) % population working outside residential area | Proximity to an urban area yielding higher mobility  Higher scores = higher mobility |

Notes: ALD: Long-term condition; (+) positively associated with (corr >0.2); (-) negatively associated with (corr <0.2)

**Dimension 1: COVID-19 related chronic diseases**

We used the proportion of patients provided with specific national health insurance coverage for long-term conditions termed “affection de longue durée” (ALD) by the French National Health Insurance services as a proxy for prevalence of chronic diseases in each department. Among the 31 conditions listed as beneficiaries of long-term coverage, 3 were selected as they were found to be significantly associated with a greater risk of COVID-19 severe illness or mortality: diabetes (type 1 and 2, these are unspecified under health insurance coverage), coronary heart disease, and severe high blood pressure. Of note, as severe high blood pressure was removed from the list of conditions ensuring specific coverage in 2011, the prevalence of patients under this condition only includes patients up to that date.

**Dimension 2: Healthcare supply**

We used the density of medical practitioners, registered nurses and pharmacists per 100,000 population in 2018 as a measure of healthcare supply, available from the Directorate for Research, Studies, Evaluation and Statistics of the French Ministry of Solidarities and Health, calculated at the department level from the directory of primary healthcare providers to which health professionals are required to subscribe to practice. PCA yielded two variables (percentage of variance explained = 81.2% and 10.1%), termed supply of primary healthcare workers, with a positive correlation to the density of all primary healthcare workers (general practitioners, nurses and pharmacists) and the supply of pharmacists (specifically positively correlated to the density of pharmacists).

**Dimension 3: Socioeconomic deprivation**

We used a validated aggregated score, the French deprivation index (FDEP), based on work by Rey and colleagues [1]. FDEP was calculated using data at the municipality level, including median household income, percentage of high school graduates in the population aged 15 years or older, the percentage of blue-collar workers in the active population and the unemployment rate. The first component of the PCA gives the score at municipal level and then is aggregated at the department level. A second indicator termed deprivation heterogeneity was computed as the variance in FDEP within a department.

**Dimension 4: Urbanization**

We used population density per km² in 2016, household overcrowding (defined if the number of rooms available for the members of the household is deemed lower than what is necessary) and number of individuals per family unit in 2016, proportion of population living in large urban areas in 2016, proportion of the active population working outside of their employment area of residence and proportion of the active population using some form of public transport in 2016. These variables were available from INSEE. PCA yielded two variables (percentage of variance explained = 88.3% and 8.4%) we termed urbanization. This composite variable is characterized by a positive correlation with over occupation of housing, percentage of the population living in an urban center, population density (log), percentage of the population using public transportation and the percentage of the population working outside the residential area; and work-related mobility, negatively correlated to the percentage of population living in an urban center and positively correlated to the percentage of population working outside its residential area.

**Box 1. Multiscale geographically weighted regression (MGWR) modelling**

GWR assumes spatial non-stationarity by including geographical coordinates of observations and in computing a local model for each location of interest (i.e., for each department *i*):

$$y_{i}=\hat{\beta}_{0\left( i \right)}+\hat{\beta}_{1\left( i \right)}X_{1\left( i \right)}+\hat{\beta}_{2\left( i \right)}X_{2\left( i \right)}+...+\hat{\beta}_{k\left( i \right)}X_{k\left( i \right)}+\varepsilon_{i}$$

where *y_i_* is the observation of the dependant variable (i.e., excess mortality) for departement *i*, *β_k(i)_* and *X_k(i)_* are the parameter estimates and the explanatory variables for the *i^th^* departement, respectively, and *ε_i_* is a random error term. Parameter estimates are computed at each location by calibrating a locally weighted regression using the following estimator in matrix form:

$$\hat{\beta}_{i}=\left( X'W_{i}X \right)^{-1}X'W_{i}y$$

where *β_i_* is an *k* x 1 vector of parameter estimates, *X* is an *n* x *k* matrix of explanatory variables, *y* is a *k* x 1 vector of observations for the dependent variable, and *W_i_* is a spatial weight matrix defined through a specific neighbouring and weighting scheme.

The neighbouring scheme can be defined as either Euclidean distance, or a nearest-neighbour, while the weighting scheme is characterized by a kernel function that can be bisquare, exponential, Gaussian, etc., the aim being to give more weight to closer observations over farther ones. Here we used an exponential kernel function involving a nearest-neighbour based neighbouring matrix. The exponential function considers all the observations in each local regression, but with a weight inversely proportional to the distance, thus avoiding losing statistical power for our relatively small sample of statistical individuals (N=96). The number of nearest neighbours is usually determined using an optimal bandwidth, selected by minimizing a corrected Akaike criterion (AICc), and then applied equally to each covariate. To overcome this limitation of applying the same bandwidth to each relationship, we used a recent extension to the GWR developed by Fotheringham and colleagues [2], called multiscale GWR (MGWR, also called conditional GWR or flexible bandwidth GWR), that computes a specific bandwidth for each covariate. The advantage of MGWR is to more accurately capture the spatial heterogeneity within and across spatial processes, thus minimizing overfitting and ultimately reducing bias in the parameter estimates [3,4].

MGWR reformulates GWR as follows:

$$y_{i}=\sum_{j=1}^{k} \beta_{bwj}\left( u_{i},v_{i} \right)x_{ij}+\varepsilon_{i}$$

Where *bwj* in $\beta_{bwj}$ depicts the bandwidth used for calibration of the *j^th^* conditional relationship [2]. To calibrate MGWR models, the authors suggest using backfitting algorithms, as commonly used for calibrating generalized additive models. Fotheringham et al. [2] explain that following the logic of GAM, the term $\beta_{bwj}\left( u_{i},v_{i} \right)$ should be defined as the *j^th^* additive term *f_j_* resulting in a GAM-style MGWR:

$$y=\sum_{j=1}^{k} f_{j}+\varepsilon$$

where *f_j_* is a smoothing function applied to the *j^th^* covariate. The model is calibrated using a backfitting algorithm that derives a set of bandwidth parameters for the *j* processes being modelled [4].

For MGWR inferences, we addressed in this research the issue of multiple hypothesis tests due to the multiple local parameters estimates. Following the recommendations of Da Silva and Fotheringham [5], we applied a correction to the α-value (usually equalling 0.05):

$$\alpha=\frac{\xi}{\frac{ENP}{k}}$$

where ξ is the desired joint type 1 error rate, ENP is the effective number of parameters and k the number of variables. In our models, we fixed ξ at 0.05 and found α = 0.013, so that local parameter estimates were considered as statistically significant when their critical t-values > |2.5|, i.e., a more conservative value than in usual ordinary least squares models for such a type 1 error rate (|1.96|).

**References**

1. Rey G, Jougla E, Fouillet A, Hémon D. Ecological association between a deprivation index and mortality in France over the period 1997 - 2001: variations with spatial scale, degree of urbanicity, age, gender and cause of death. BMC Public Health. 2009;9:33.

2. Fotheringham AS, Yang W, Kang W. Multiscale Geographically Weighted Regression (MGWR). Ann Am Assoc Geogr. Taylor & Francis; 2017;107:1247–65.

3. Wolf LJ, Oshan TM, Fotheringham AS. Single and Multiscale Models of Process Spatial Heterogeneity. Geogr Anal. 2018;50:223–46.

4. Oshan TM, Smith JP, Fotheringham AS. Targeting the spatial context of obesity determinants via multiscale geographically weighted regression. Int J Health Geogr. 2020;19:11.

5. Silva AR da, Fotheringham AS. The Multiple Testing Issue in Geographically Weighted Regression. Geogr Anal. 2016;48:233–47.
